# Heat Hyperalgesia and Mechanical Hypersensitivity Induced by Calcitonin Gene-Related Peptide in a Mouse Model of Neurofibromatosis

**DOI:** 10.1371/journal.pone.0106767

**Published:** 2014-09-03

**Authors:** Stephanie White, Blanca Marquez de Prado, Andrew F. Russo, Donna L. Hammond

**Affiliations:** 1 Department of Anesthesia, University of Iowa, Iowa City, Iowa, United States of America; 2 Department of Molecular Physiology and Biophysics, University of Iowa, Iowa City, Iowa, United States of America; 3 Department of Pharmacology, University of Iowa, Iowa City, Iowa, United States of America; University of Würzburg, Germany

## Abstract

This study examined whether mice with a deficiency of neurofibromin, a Ras GTPase activating protein, exhibit a nociceptive phenotype and probed a possible contribution by calcitonin gene-related peptide. In the absence of inflammation, *Nf1^+/−^* mice (B6.129S6 Nf1<tm1Fcr>/J) and wild type littermates responded comparably to heat or mechanical stimuli, except for a subtle enhanced mechanical sensitivity in female *Nf1^+/−^* mice. Nociceptive phenotype was also examined after inflammation induced by capsaicin and formalin, which release endogenous calcitonin gene-related peptide. Intraplantar injection of capsaicin evoked comparable heat hyperalgesia and mechanical hypersensitivity in *Nf1^+/−^* and wild type mice of both genders. Formalin injection caused a similar duration of licking in male *Nf1^+/−^* and wild type mice. Female *Nf1^+/−^* mice licked less than wild type mice, but displayed other nociceptive behaviors. In contrast, intraplantar injection of CGRP caused greater heat hyperalgesia in *Nf1^+/−^* mice of both genders compared to wild type mice. Male *Nf1^+/−^* mice also exhibited greater mechanical hypersensitivity; however, female *Nf1^+/−^* mice exhibited less mechanical hypersensitivity than their wild type littermates. Transcripts for calcitonin gene-related peptide were similar in the dorsal root ganglia of both genotypes and genders. Transcripts for receptor activity-modifying protein-1, which is rate-limiting for the calcitonin gene-related peptide receptor, in the spinal cord were comparable for both genotypes and genders. The increased responsiveness to intraplantar calcitonin gene-related peptide suggests that the peripheral actions of calcitonin gene-related peptide are enhanced as a result of the neurofibromin deficit. The analgesic efficacy of calcitonin gene-related peptide receptor antagonists may therefore merit investigation in neurofibromatosis patients.

## Introduction

Neurofibromatosis 1 (NF1) is an autosomal dominant disorder that results in reduced levels of neurofibromin, a GTPase activating protein (GAP) involved in the regulation of Ras signaling (i.e. a Ras-GAP). This genetic disorder affects one in 3500 births worldwide – an incidence that equates to ∼90,000 Americans and a million persons worldwide [1]–[4]. Nearly half of these cases result from new mutations. As such, *Nf1* has one of the highest rates of new mutations for any known single gene disorder [3], [5]. One in four individuals with NF1 experience chronic bodily pain, as well as migraine and headache pain, over periods of months to years [1], [6], [7]. Severe pain also results from neurofibromas on spinal roots and malignant peripheral nerve sheath tumors [3]. The chronic nature of the pain, as well as its lancinating and paroxysmal character, contribute to the poor quality of life for patients with NF [8]. There is a great need for mechanistic based pharmacotherapies for the relief of pain in this patient population.

Early studies by Hingtgen and colleagues focused attention on the possible role of calcitonin gene-related peptide (CGRP) in pain associated with NF1. CGRP is a key factor in peripheral inflammation and in the production of nociception both in the spinal cord and in the periphery [9]–[12]. Interestingly, high densities of CGRP-immunoreactive fibers are present in neurofibromas in patients [13]. Using a rodent model of NF1 (*Nf1* ^+/−^ mice), Hingtgen and colleagues determined that haploid insufficiency of neurofibromin is associated with an increased release of CGRP from cultured dorsal root ganglion (DRG) neurons and spinal cord slices [14], as well as increased excitability of primary afferent neurons that convey pain [15], [16]. More recent studies determined that the DRG of *Nf1^+/−^* mice have increased transcripts for Nav1.7 and Nav1.8, which are likely to underlie the enhanced excitability of primary afferent neurons [17], as well as increased N-type calcium currents that could contribute to enhanced release of neurotransmitter [18]. Collectively, these data suggest that an increased release of CGRP and decreased thresholds for activation of sensory neurons may underlie pain in NF1.

The aim of this study was to determine whether *Nf1^+/−^* mice exhibit a nociceptive phenotype and could serve as a model of pain in NF1, potentially facilitating the development of new therapies. Nociceptive responsiveness of male and female *Nf1^+/−^* mice to heat and mechanical stimuli was determined in the absence of inflammation and after inflammation induced by exogenous administration of CGRP or by formalin or capsaicin, two agents that can release CGRP in the spinal cord and periphery. Ancillary studies quantitated levels of transcript for CGRP in the DRG and receptor activity-modifying protein-1 (RAMP1) in the spinal cord. RAMP1 associates with the calcitonin-like receptor to form the CGRP receptor [19], and is rate limiting for the activity of CGRP [20]. The results indicate that *Nf1^+/−^* mice are not a robust animal model for the pain experienced by NF patients, a conclusion that was also reached by O'Brien et al. [21]. Nonetheless, the finding that neurofibromin deficit is associated with an enhanced effect of CGRP in the periphery suggests that CGRP receptor antagonists may merit investigation for the treatment of pain in NF patients.

## Materials and Methods

### Experimental model

Mutation of *Nf1* in one allele is sufficient for expression and nearly complete penetrance of the disorder in humans. These studies therefore used *Nf1^+/−^* mice as a rodent model of NF1. These mice exhibit an increased predisposition to develop tumors as they age [22], [23]. Use of homozygous null mutant mice was not feasible as the mutation is embryonic lethal [22], [24]. Moreover, no definitive examples of patients homozygous for mutation of *Nf1* have been identified [25].

Litter-matched wild type (WT) and *Nf1^+/−^* (B6.129S6 Nf1<tm1Fcr>/J) mice of both genders (Jackson, Bar Harbor, Maine) weighing 23–30 grams were used. Female mice were tested randomly throughout the estrous cycle. Mice were housed in groups of five on SoftZorb paper enrichment bedding in a temperature controlled room on a 12-hr light/dark cycle. All testing occurred during the light cycle from 9:00 to 15:00 hr. Mice were euthanized at the conclusion of the experiments by CO_2_ inhalation. These experiments were approved by the University of Iowa Animal Care and Use Committee (ACURF 0711237) and were conducted in accordance with the guidelines of the National Institutes of Health Guide for the Care and Use of Laboratory Animals and the guidelines of the International Association for the Study of Pain. With the exception of the capsaicin test, mice were used once and received only one dose of an agent. Every effort was made to minimize the number of mice used and their suffering. In all experiments, the investigator was blinded to the genotype. Blinding to intraplantar (ipl) treatment was not possible because capsaicin and formalin both produce spontaneous pain behaviors, while CGRP produces erythema and inflammation upon injection.

### Heat threshold

Experiments were conducted to determine responsiveness to noxious heat, and whether heat hyperalgesia produced by ipl injection of CGRP or capsaicin differed by genotype or gender. Mice were acclimated to the testing environment for several hrs on two successive days and then allowed to move freely for one hr within a small Plexiglas enclosure that rested on an elevated glass surface maintained at 25°C. On the third day, the mice were acclimated to the environment for two more hrs and again allowed to acclimate to the testing chambers for another 30 min. A high intensity beam of light was then positioned under each hindpaw, and the latency to withdraw the hindpaw from the heat stimulus was recorded (UARD group, La Jolla, CA). If the mouse did not withdraw its paw within 20 sec, the test was terminated to prevent tissue injury. Care was taken to ensure that the surface of the hindpaw was flush with glass to avoid a heat-sink effect [26].

The first set of experiments assessed basal sensitivity to a noxious heat stimulus. The radiant heat stimulus was delivered at two different rates, 2.5°C/sec and 6.5°C/sec, as measured at the surface. These rates corresponded to 0.6°C/sec and 1.0°C/sec, as measured within the tissue. For both stimuli, the subdermal temperature at which withdrawal occurred was 40–41°C, in agreement with similar studies in the rat [27]–[29]. The lower rate of heating, which likely preferentially activates C-fibers, was used in the second set of experiments that assessed heat hyperalgesia. After determination of baseline paw withdrawal latency, an ipl injection of 5 µg CGRP, 0.01% capsaicin, or PBS was made into a hind paw. Paw withdrawal latency was redetermined 15, 30, 45, and 60 min after CGRP and 15 min after capsaicin.

Data were expressed as the mean ± S.E.M. Baseline paw withdrawal latencies were compared using a two-way analysis of variance (ANOVA) in which genotype was one factor and intensity was the second factor. The effects of CGRP or capsaicin were compared to their vehicle using a two-way repeated measures ANOVA in which one factor was genotype and the repeated factor was time. The Holm-Sidak test was used to make comparisons among mean values for the different treatment groups. A P<0.05 was considered significant in these and all subsequent analyses.

### Mechanical threshold

Additional experiments determined mechanical threshold and examined whether mechanical hypersensitivity after ipl injection of CGRP or capsaicin differed by genotype or gender. Mice were acclimated to the testing environment for several hrs on two successive days. On the third day, the mice were acclimated for two more hrs to the environment and then allowed to move freely for a further 30 min within a small Plexiglas enclosure with a mesh floor. Mechanical hypersensitivity was assessed with a series of von Frey filaments that ranged from 1.65–4.31 log mN (0.0008–2 g). The filaments were applied to the plantar surface of each hindpaw between the pads. Care was taken to vary the site of application in this area. Testing was initiated by a two-sec application of the 3.84 filament before and the 3.22 filament after ipl injection of CGRP or capsaicin with enough pressure to cause the filament to bend slightly. If the mouse withdrew or lifted the paw, filaments of successively lower force were applied until one that produced no response to five applications was identified. The next higher force filament was then applied for a total of five times and the percentage of responses was recorded for that filament. Filaments of successively higher force were applied in this manner until a filament was reached that produced withdrawal on all five presentations or the 4.31 filament was reached. Higher filaments were not used because they lifted the hindpaw before the filament bent. After determination of basal threshold, either 5 µg CGRP, 0.001 or 0.01% capsaicin or their respective vehicles were injected. Mechanical hypersensitivity was redetermined 5 and 15 min after CGRP or PBS, and 15 min after capsaicin or vehicle by which time capsaicin's associated spontaneous pain behaviors had subsided.

Force-response curves were generated for both hindpaws and fit by non-linear regression to determine an EF_50_ with 95% confidence limits (CL) using Graphpad Prism. The minimum and maximum values were constrained to 0 and 100, respectively. Comparisons of EF_50_ values were made by F-test using Graphpad Prism version 5.0.

### Spontaneous pain behavior

Spontaneous pain behaviors evoked by ipl injection of 0.01% capsaicin or 2% formalin in one hindpaw were also quantified. In the case of capsaicin, the amount of time spent licking the injected paw was recorded in 5 min epochs for 15 min; spontaneous pain behaviors were essentially absent after 15 min. In the interest of making efficient use of each mouse and because the licking evoked by capsaicin was highly variable, each mouse received an injection of capsaicin in the left and right hindpaw, separated by a month. The duration of licking of each hindpaw was then averaged for that mouse. In the case of formalin, spontaneous pain behaviors were recorded for 90 min with a video camera and analyzed offline. The amount of time spent licking the paw, as well as the percentage of mice that exhibited guarding, flinching or unweighting of the injected paw, were quantified in 5 min epochs.

Duration of licking after capsaicin was expressed as the mean ± S.E.M. and compared between genotypes by two-way ANOVA for repeated measures. The Holm-Sidak test was used to compare group mean values. The duration of licking after formalin was analyzed in the same manner. In addition, the total time spent licking during phase one (0–5 min), phase two (15–50 min) and phase three (55–90 min) of the formalin test was calculated and compared by two-way ANOVA for repeated measures. Finally, guarding, flinching or unweighting of the injected paw after formalin were collectively scored as either present or absent. The percentage of mice that exhibited these behaviors was averaged over time for each of the three phases of the formalin test and compared by ANOVA.

### Quantitation of CGRP and RAMP1 mRNA

Mice were euthanized by CO_2_ inhalation followed by decapitation. The entire spinal cord and all DRG were rapidly removed from each mouse and stored in RNAlater (Ambion, Austin, TX). Total RNA was isolated from the tissue using the RNeasy kit according to the manufacturer's directions (Qiagen, Valencia, CA) and DNA contamination removed by digestion with Amp Grade DNase I (Invitrogen, Carlsbad, CA). Briefly, less than 30 mg of tissue was disrupted and homogenized in RTL buffer containing β-mercaptoethanol using a Tissue Tearor. The lysate was then centrifuged and the supernatant saved in a clean tube. The RNA was precipitated using 1 volume of 70% ethanol and loaded on the column. After one wash, 40 µl of buffer containing 1 unit DNase I was applied to the column and allowed to incubate for 15 min. The DNase was then removed by extensive washing. The RNA was eluted using RNase free water, and RNA concentration was determined by spectrometry. The RNA integrity number was determined for a subset of samples, and ranged between 8.1 and 8.6. Reverse transcription was performed using the Taqman RT-PCR mix (Applied Biosystems, Carlsbad, CA) with 0.5 µg RNA, 1X RT buffer, 5.5 mM MgCl_2_, 0.5 mM dNTP, 4 units RNase inhibitor, 2.5 µM random hexamers, 12.5 units Multiscribe reverse transcriptase in 10 µl at 25°C for 10 min, 48°C for 30 min and 95°C for 5 min. Q-PCR was performed using 50 ng cDNA, 670 nM each primer (67 nM for CGRP primers) and 1X IQ^tm^ SYB Green Supermix (BioRad, Hercules, CA) in 15 µl. The cycle conditions were: 95°C for 10 min, followed by 40 cycles of 95°C for 15 s and annealing/extension at 60°C for 30 s and 72°C for 45 sec. Reactions were performed in triplicate and analyzed using a Bio-Rad MY-IQ thermocycler. At the end of amplification, a thermal melt curve was generated. The one sample that that did not yield a homogenous melt curve was excluded. Primers for mRAMP1, CGRP, mGAPDH and β-actin have been described [20]. During this work, it was discovered that the description of the reverse primer for mRAMP1 in Zhang et al. (2007) contained a typographical error and an extraneous nucleotide. Although the correct primer (5′GCACTTGCTGAAGTATCGATGG3′) was used for that work, the textual errors were replicated when ordering primer for this study. Nonetheless, the PCR product yielded the correct cDNA as verified by sequence analysis. Cycle thresholds (C_T_) for mRAMP1, CGRP, GAPDH and β-actin were converted to absolute numbers using standard curves generated with serial dilutions of pGEM-QmRAMP1, pQmCGRP, pQmGAPDH and pQmβ-actin plasmids, respectively. For RAMP1, the mean efficiency of the PCR was 99.1±1.1% with a mean slope of −3.35±0.03. For β-actin, the mean efficiency was 94.8±1.0% with a mean slope of −3.43±0.03. For CGRP, the efficiency of the PCR was 93.2% with a mean slope of −3.299 and for GAPDH the efficiency of the PCR was 101.0% with a mean slope of −3.496.

### Drugs and Vehicle Controls

All drugs were purchased from (Sigma, St Louis, MO) and prepared fresh each day. Calcitonin gene-related peptide was dissolved in PBS, which served as its vehicle control. Capsaicin was dissolved in 5% Tween 80 and 5% ethanol and brought to volume with PBS. In the von Frey experiments, a few mice received PBS as the control and others received PBS with 5% Tween 80 and 5% ethanol as the control. As there was no difference in the effects of either vehicle, the data were pooled for statistical analysis. Formalin was diluted to 2% using PBS. All drugs were injected in the plantar surface of one hindpaw with a 33-gauge stainless steel injector needle. Formalin was injected in a volume of 20 µl, while capsaicin or CGRP was injected in a volume of 10 µl. Drug delivery was monitored by following the movement of an air bubble in the tubing that connected the injector to the syringe pump.

## Results

### Nociceptive thresholds in the absence of injury

**Figure 1:**
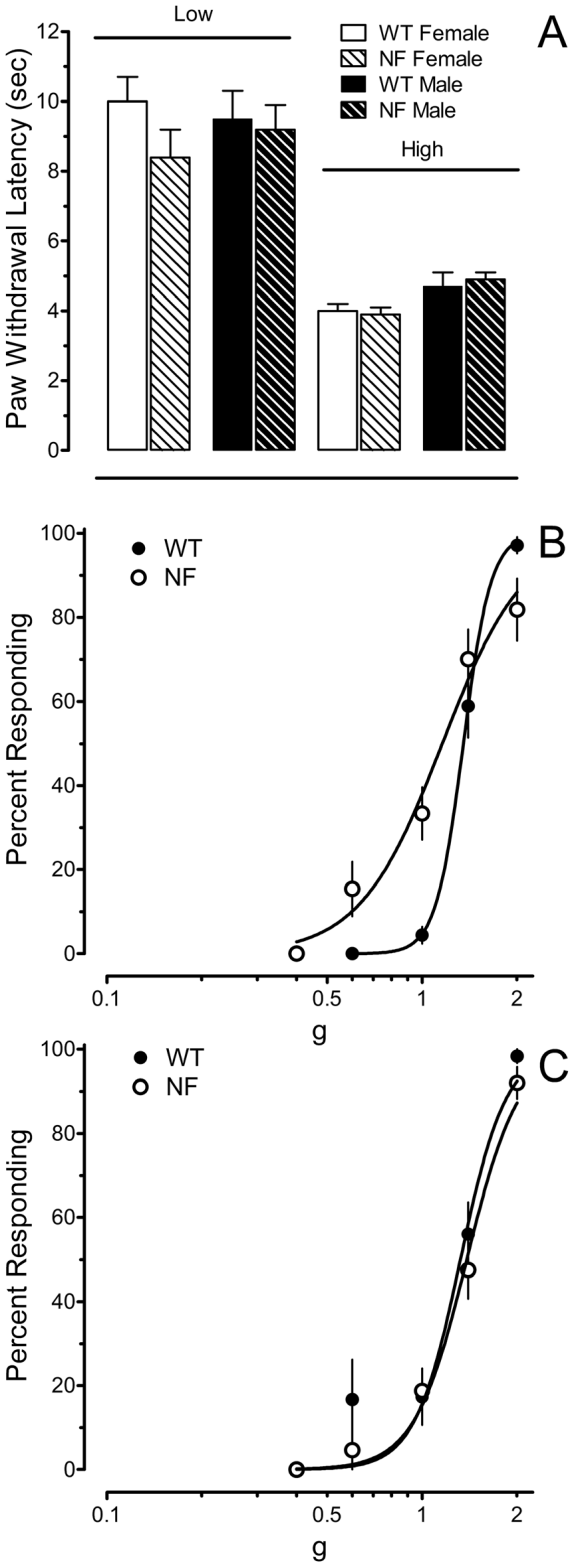
Assessment of acute nociception in *Nf1* ^+/−^ mice and WT littermates. In the absence of injury, *Nf1^+/−^* mice did not exhibit robust differences in sensitivity to noxious heat or tactile stimuli applied to the plantar surface of the hindpaw. (A) Paw withdrawal latency to low or high heating rates did not differ among genotypes or genders. Response latencies for both hindpaws were averaged to yield a single value for each mouse. Data are mean ± SEM of 6–8 mice. (B) Mechanical sensitivity was slightly greater in female *Nf1^+/−^* mice than wild type (WT) littermates (N = 18 in each group). (C) Male *Nf1^+/−^* and WT mice did not differ in their mechanical sensitivity (n = 16 and 15, respectively). Panels B and C were constructed using baseline data for the ipsilateral hindpaw of mice in the capsaicin and CGRP treatment groups. Data are expressed as the mean ± SEM.

No differences in paw withdrawal latency were evident between the genotypes or by gender at either heating rate (Fig. 1A). With respect to mechanical sensitivity, the EF_50_ (95% CL) values for female WT and *Nf1^+/−^* mice were 1.35 (1.30−1.40) g and 1.16 (1.04–1.28) g, respectively (P<0.01). The small, but significant increase in mechanical sensitivity of female *Nf1^+/−^* mice could be attributed to an enhanced responsiveness to lower force filaments (Fig. 1B). The EF_50_ (95% CL) values for male WT and male *N1f^+/−^* mice were 1.32 (1.23−1.42) and 1.38 (1.30–1.48) g, respectively (P>0.5; Fig. 1C). Thus, with the exception of a small increase in mechanical sensitivity in female *Nf1^+/−^* mice, responsiveness to heat and mechanical stimuli did not differ between the genotypes or between the genders. Subsequent experiments examined whether *Nf1* ^+/−^ mice differed from their WT littermates in their responses to inflammatory agents.

### Heat hyperalgesia evoked by capsaicin or CGRP

**Figure 2:**
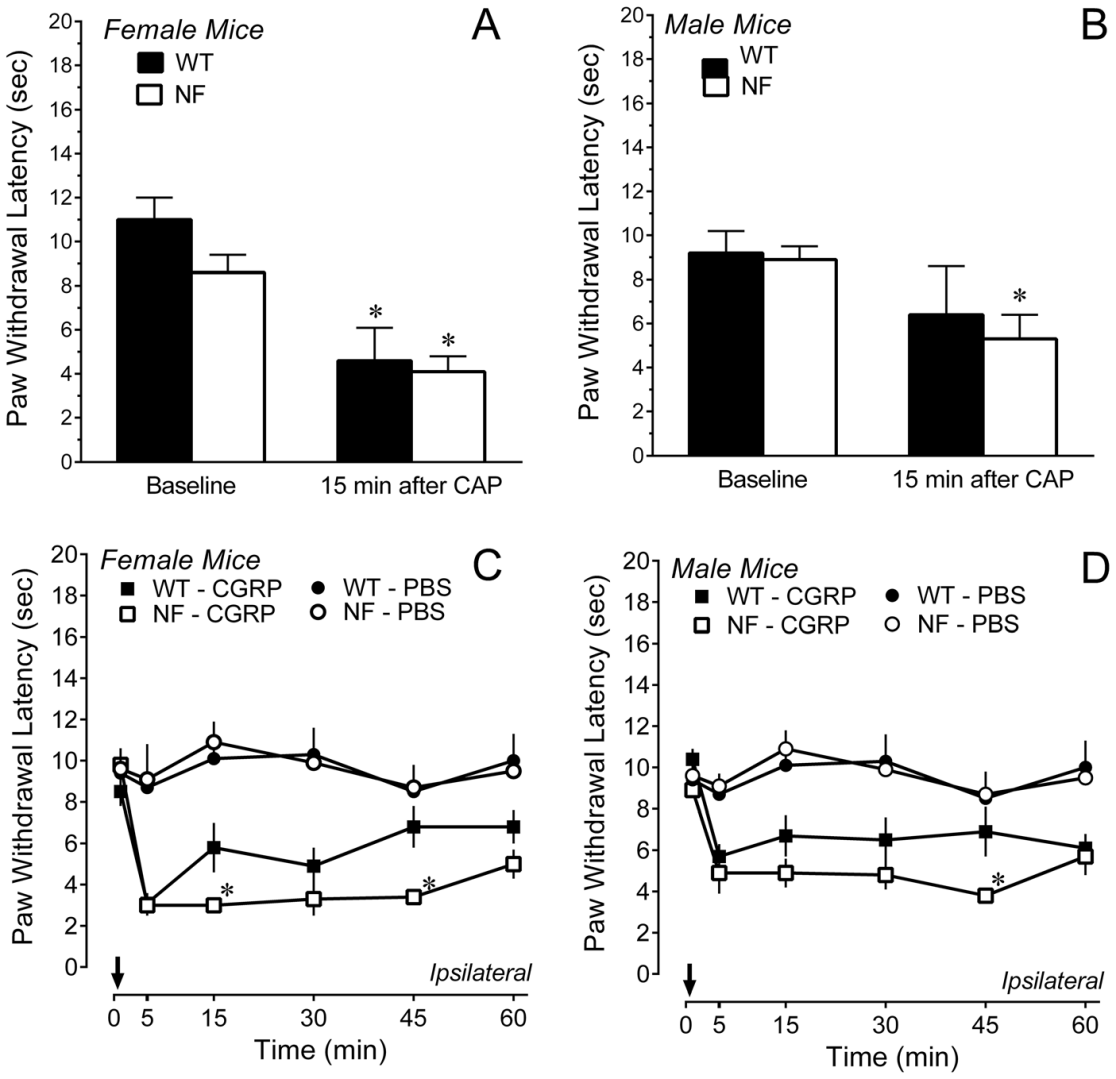
Intraplantar injection of 0.01% capsaicin or 5 µg CGRP induced heat hyperalgesia. (A, B) The magnitude of heat hyperalgesia determined 15 min after injection of capsaicin did not differ between genotypes in either females or males. * P<0.05 compared to baseline. (C, D) Paw withdrawal latency was decreased within 5 min of injection (arrow) of CGRP, but not PBS in females and males of both genotypes. Heat hyperalgesia in female and male *Nf1^+/−^* mice was greater than their WT littermates. The data for PBS are the pooled responses of two female and two male mice of each genotype. * P<0.05 compared to WT at the same time point. Data in all panels are expressed as the mean ± SEM of determinations in 6–8 mice of each gender and genotype.

Injection of 0.01% capsaicin in the hindpaw decreased paw withdrawal latency to a similar extent in female *Nf1* ^+/−^ and WT mice (Fig. 2A). Capsaicin also significantly decreased paw withdrawal latency in male *Nf1^+/−^* mice, but did not significantly decrease paw withdrawal latency in male WT mice (P = 0.13; Fig. 2B). Nonetheless, male WT and *Nf1* ^+/−^ mice had comparable paw withdrawal latencies after capsaicin (P>0.5). These results indicate that the magnitude of capsaicin-induced heat hyperalgesia was comparable in both genotypes and genders.

Intraplantar injection of CGRP produced heat hyperalgesia in *Nf1* ^+/−^ mice and WT littermates of both genders. Female *Nf1* ^+/−^ mice exhibited a significantly greater decrease in paw withdrawal latency than female WT mice 15 and 45 min after injection (Fig. 2C). Male *Nf1^+/−^* mice exhibited a significantly greater decrease in paw withdrawal latency than male WT mice 45 min after injection (Fig. 2D). Intraplantar injection of PBS did not alter paw withdrawal latency of the ipsilateral hindpaw of either gender or genotype. Thus, unlike the heat hyperalgesia induced by capsaicin, the heat hyperalgesia induced by CGRP was greater in both male and female *Nf1^+/−^* mice compared to their corresponding WT controls.

### Mechanical sensitivity after capsaicin or CGRP

**Figure 3:**
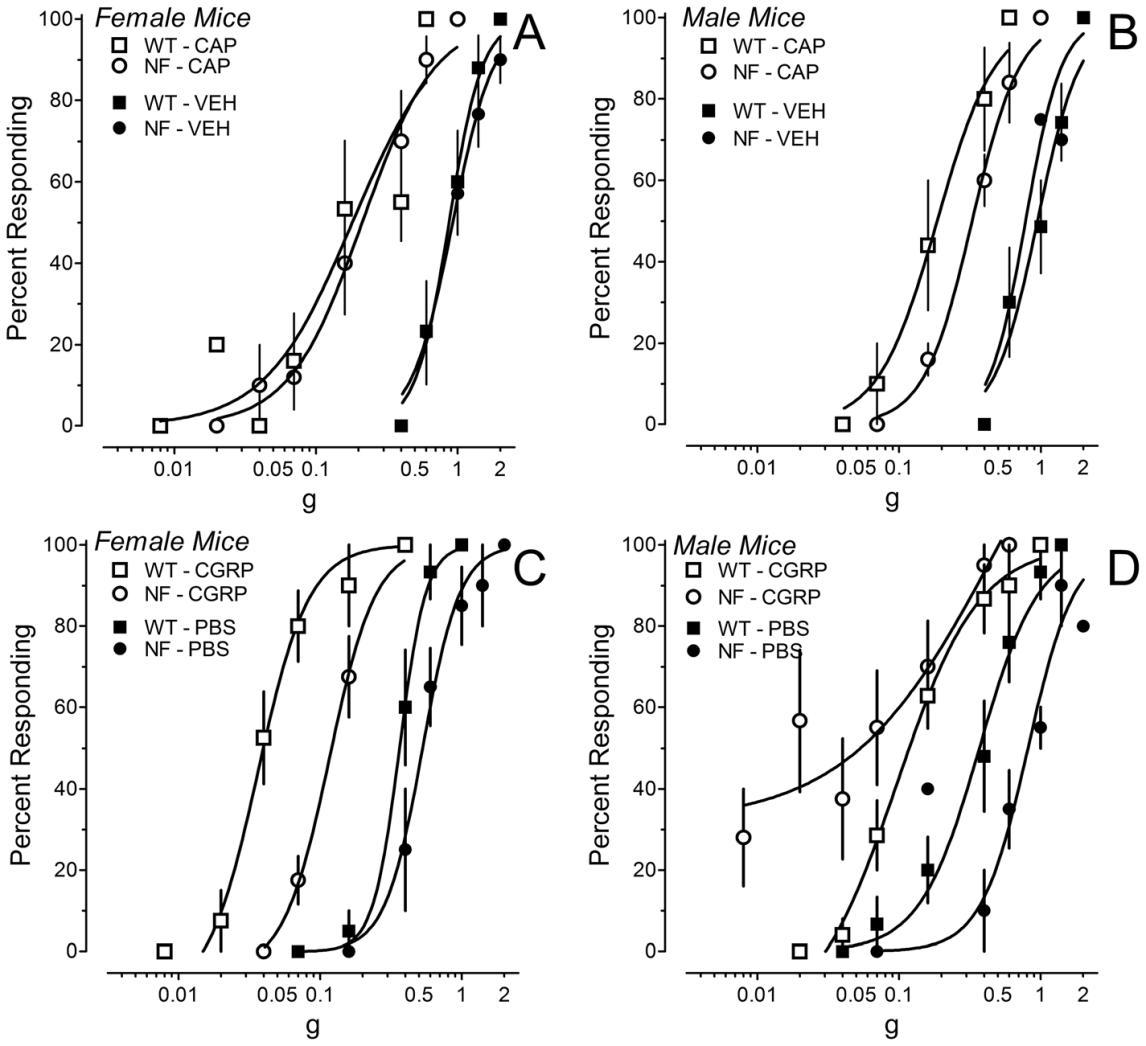
Mechanical hypersensitivity induced by intraplantar injection of 0.001% capsaicin (CAP) or 5 µg CGRP. (A, B) Force-response curves for the ipsilateral hindpaw after injection of CAP or vehicle (VEH) in *Nf1^+/−^* (circles) or WT (squares) mice. Data are the mean ± SEM of responses by 5–7 mice of each gender and genotype. (C, D) Force-response curves for the ipsilateral hindpaw after injection of CGRP or PBS in *Nf1^+/−^* (circles) or WT (squares) mice. Data in panels C-D are the mean ± SEM of responses by 4–8 mice of each gender and genotype, with the exception of CGRP in *Nf1^+/−^* mice where 11 mice were tested.

Injection of 0.01% capsaicin in the hindpaw dramatically shifted the force-response curves to the left such that the lowest filament (0.008 g, 1.65 log mN) elicited responses on more than 50% of its presentations in most mice. Although mechanical hypersensitivity did not differ between genotypes or gender, a ceiling effect could have disguised subtle differences. Fifteen min after injection of a ten-fold lower dose of capsaicin (0.001%) female *Nf1^+/−^* and WT mice exhibited equivalent mechanical hypersensitivity, while male *Nf1^+/−^* mice exhibited less mechanical hypersensitivity than male WT mice (Fig. 3B, Table 1). Injection of vehicle also produced a very small, but statistically significant leftward shift in the force-response curves compared to baseline (Table 1). Thus, in female mice, capsaicin induced an equivalent mechanical hypersensitivity in both genotypes, whereas male *Nf1^+/−^* mice were less affected.

**Table 1:** Capsaicin evokes mechanical hypersensitivity in both wildtype and *Nf1^+/−^* mice.

Female	WT		*Nf1* ^+/−^	
	Vehicle [6]	Capsaicin [6]	Vehicle [7]	Capsaicin [5]
Baseline	1.43 (1.37–1.50)	1.37 (1.31–1.44)	1.57 (1.46–1.69)	1.52 (1.29–1.79)
After	0.87 (0.73–1.03)‡	0.18 (0.11–0.29)‡ **	0.92 (0.77–1.10)‡	0.21 (0.15–0.29)‡ **
**Male**	**WT**		***Nf1*** ^+/−^	
	**Vehicle [6]**	**Capsaicin [5]**	**Vehicle [4]**	**Capsaicin [4]**
Baseline	1.42 (1.36–1.48)	1.40 (1.29–1.52)	1.51 (1.40–1.63)	1.49 (1.36–1.63)
After	0.95 (0.78–1.15)‡	0.18 (0.12–0.27)‡ **	0.78 (0.60–1.01)‡	0.32 (0.28–0.39)‡ ** a

EF_50_ and 95% confidence limits (g) of force–response curves for the ipsilateral hindpaw before and 15 min after intraplantar injection of 0.001% capsaicin or vehicle.

Curves were fit by non–linear regression with minimum constrained to 0 and maximum to 100. Numbers of mice in each group appear in brackets.

*P<0.05,

** P<0.01 compared to vehicle at corresponding time point.

† P<0.05,

‡ P<0.01 compared to baseline value.

a P<0.05 compared to WT at corresponding time point.

Intraplantar injection of CGRP or PBS also produced mechanical hypersensitivity of the ipsilateral hindpaw. Five min after ipl injection of PBS or 5 µg CGRP, force-response curves for the ipsilateral paw were shifted left in both genotypes and genders (data not shown). The shift by CGRP was much greater than that by PBS (data not shown). Fifteen min later, the shift in force-response curves by PBS was greatly diminished but still significantly to the left of baseline values (Table 2 and Fig. 3C, D). In contrast, the dramatic leftward shift produced by CGRP was sustained through 15 min (Table 2). In female *N1f^+/−^* mice, CGRP induced less mechanical hypersensitivity than female WT mice (Fig. 3C). However, male *Nf1^+/−^* mice exhibited greater mechanical hypersensitivity than male WT mice at very low filament forces (Fig. 3D). These results indicate that peripheral injection of CGRP produced mechanical hypersensitivity in both genotypes, and that the magnitude was a function of gender.

**Table 2:** Calcitonin gene-related peptide evokes mechanical hypersensitivity in both wildtype and Nf1^+/−^ mice.

Female	WT		*Nf1* ^+/−^	
	PBS [4]	CGRP [8]	PBS [4]	CGRP [8]
Baseline	1.51 (1.38–1.67)	1.25 (1.16–1.35)	1.17 (0.86–1.59)^a^	0.98 (0.88–1.05)^aa^
After	0.36 (0.29–0.45)‡	0.04 (0.03–0.05)‡ **	0.53 (0.44–0.64)‡ ^aa^	0.12 (0.10–0.15)‡ ** aa
**Male**	**WT**		***Nf1*** ^+/−^	
	**PBS [5]**	**CGRP [8]**	**PBS [5]**	**CGRP [11]**
Baseline	1.38 (1.16–1.65)	1.22 (1.07–1.41)	1.40 (1.27–1.55)	1.29 (1.18–1.41)
After	0.37 (0.28–0.48)‡	0.12 (0.10–0.16)‡ **	0.82 (0.66–1.00)‡ ^aa^	0.04 n.d.

EF_50_ and 95% confidence limits (g) determined for force-response curves for the ipsilateral hindpaw before and 15 min after intraplantar injection of 5 µg CGRP or PBS

Curves were fit by non-linear regression with minimum constrained to 0 and maximum to 100. Numbers of mice in each group appear in brackets.

*P<0.05,

** P<0.01 compared to vehicle at corresponding time point.

† P<0.05,

‡ P<0.01 compared to baseline value.

a P<0.05,

aa P<0.01 compared to WT at corresponding time point.

### Spontaneous behavior after acute inflammatory stimuli

**Figure 4:**
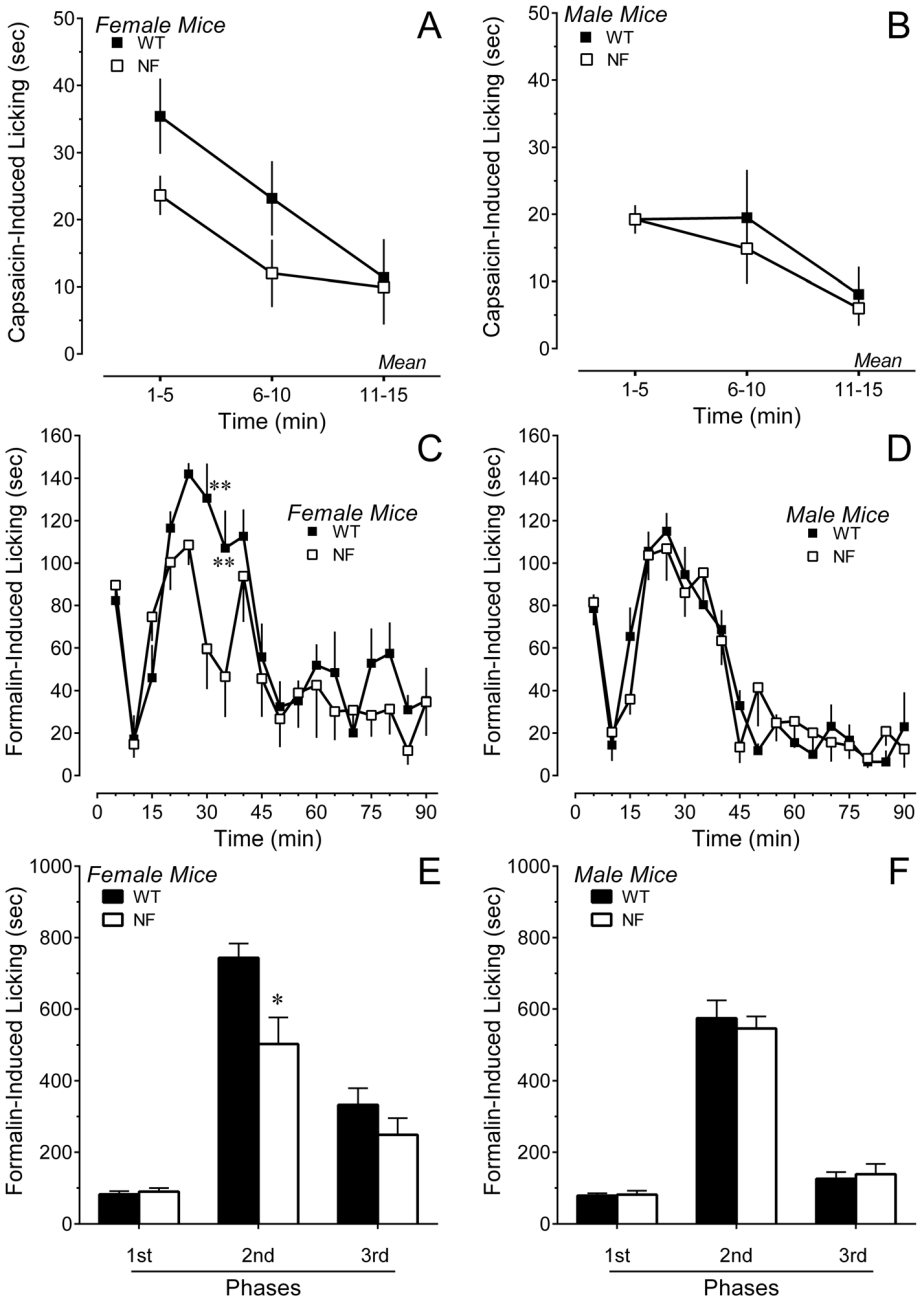
Duration of licking of the ipsilateral hindpaw after intraplantar injection of inflammatory irritants. Panels A and B illustrate the time course of licking after intraplantar injection of 0.01% capsaicin in the hindpaw of female or male mice, respectively. Panels C and D illustrate the time course of licking after intraplantar injection of 2% formalin in female or male mice, respectively. Panels E and F present the total duration of licking in phases 1 (0–5), 2 (15–55 min) and 3 (55–90 min) of the formalin test in female and male mice, respectively. Data are the mean ± S.E.M. of determinations in 6–8 mice of each gender and genotype. * P<0.05, ** P<0.01 compared to corresponding WT littermates.

Injection of inflammatory agents such as capsaicin and formalin produced a variety of spontaneous pain behaviors. In the case of 0.01% capsaicin, the predominant behavior measured in the first 15 min was licking of the hindpaw. Female *Nf1^+/−^* and WT mice did not differ statistically in the duration of licking (simple main effect for genotype: P = 0.066; Fig. 4A). Male mice of each genotype exhibited comparable durations of licking (P>0.6; Fig. 4B). Similar conclusions were reached when the data were converted to area under the curve for the 15 min period. Thus, a deficit in neurofibromin neither facilitated nor inhibited capsaicin-induced spontaneous pain behaviors.

**Figure 5:**
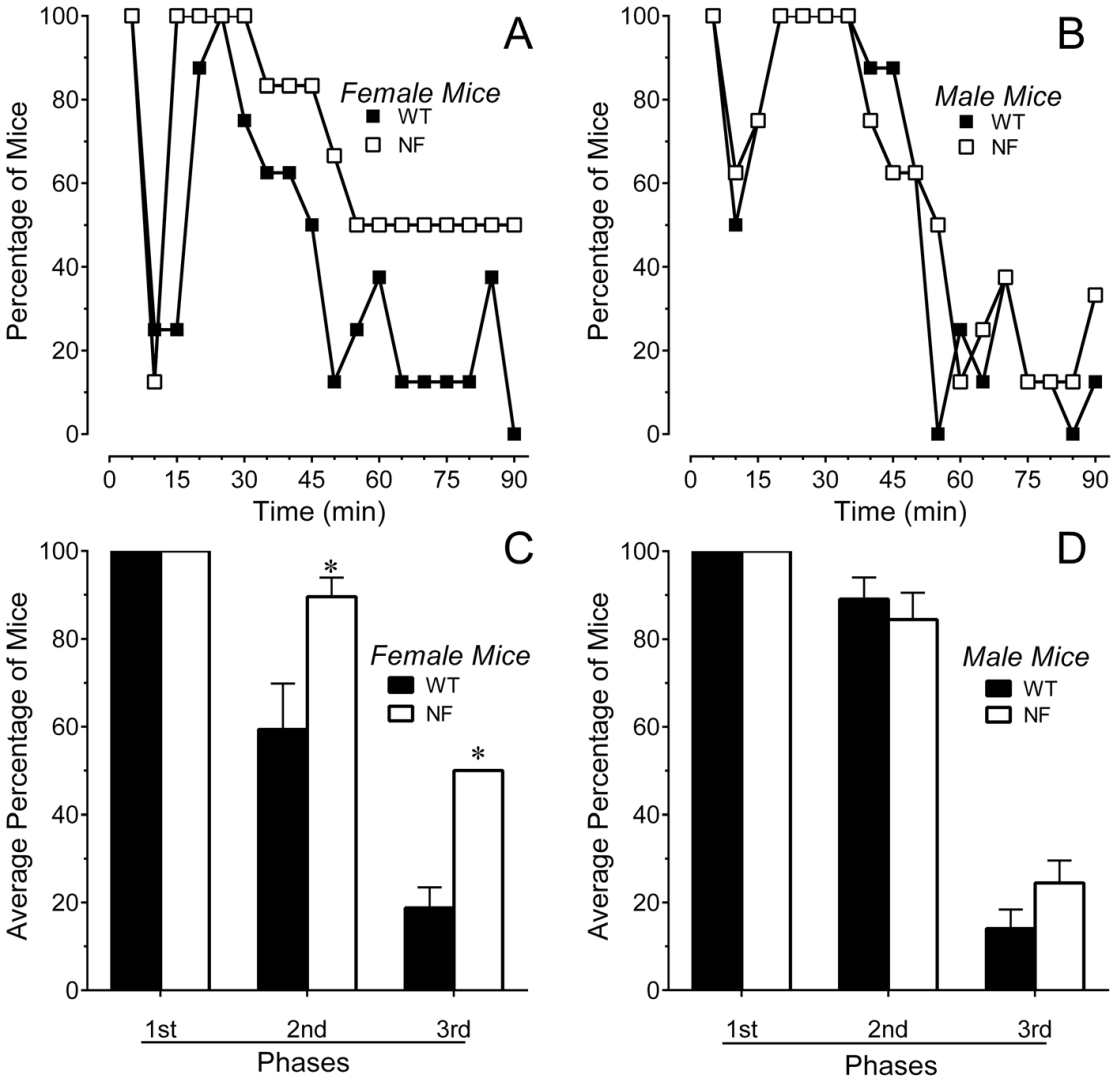
Intraplantar injection of formalin induced spontaneous pain behaviors in both genders and genotypes. Time course of guarding, flinching or unweighting of the hindpaw after intraplantar injection of formalin in (A) female or (B) male mice. The percentage of female *Nf1^+/−^* mice that exhibited these behaviors was higher than WT littermates, but did not differ in male mice of either genotype. Percentage of (C) female or (D) male mice exhibiting guarding, flinching or unweighting of the hindpaw averaged for the first phase (0–5 min), second phase (15–55 min) and third phase (55–90 min) of the formalin test. Six to eight mice of each gender and genotype were tested. * P<0.05, ** P<0.01 compared to corresponding WT littermate.

In the case of formalin, a constellation of behaviors was observed over 90 min that included guarding behaviors and unweighting of the affected hindpaw, as well as licking or flinching of the ipsilateral hindpaw. Interestingly, the duration of licking in female *Nf1^+/−^* mice was significantly lower in the second phase compared to female WT mice (Fig. 4C, E). There was no difference in the time spent licking in male mice of either genotype (Fig. 4D, F). Analysis of guarding, unweighting and flinching behaviors revealed that female *Nf1^+/−^* mice exhibited a much higher incidence of these behaviors than female WT mice during the second and third phase (Fig. 5A). These behaviors are likely to have interfered with licking. There was no significant difference in the incidence of these behaviors between male *Nf1^+/−^* and WT mice (Fig. 5B, D). Formalin increased paw thickness by the same extent in all genotypes and genders (by 0.8±0.1, 0.9±0.1, 0.9±0.1 and 0.7±0.1 mm, n = 8 each group; P>0.5). Contralateral paw thickness ranged between 1.3 and 1.4 mm.

### CGRP and RAMP1 Transcripts

**Figure 6:**
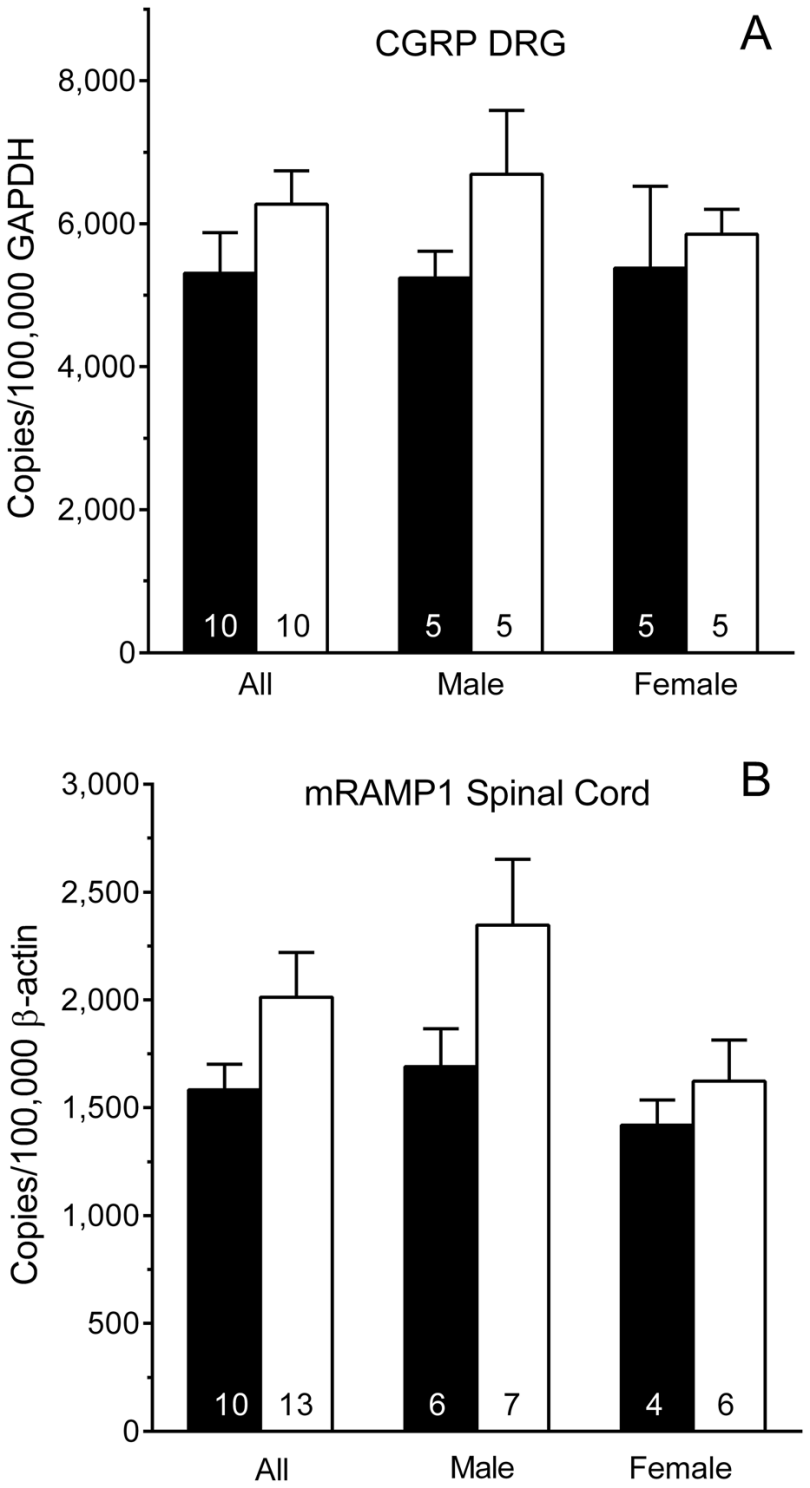
Levels of CGRP mRNA and RAMP1 mRNA. (A) Levels of CGRP mRNA in the dorsal root ganglion (DRG) of *Nf1^+/−^* and WT mice did not differ by genotype or gender. (B) Levels of RAMP1 mRNA in the spinal cord of *Nf1^+/−^* and WT mice did not differ by gender or genotype. Data are expressed as mean ± SEM copies normalized to 100,000 GAPDH for the DRG and to 100,000 β-actin for spinal cord. Numbers in the columns are the number of mice. * P<0.05 compared to corresponding WT littermates. *Nf1^+/−^* mice (open bars); WT mice (solid bars)

Figure 6A illustrates levels of CGRP mRNA in the DRG of *Nf1^+/−^* mice and their WT littermates. There were no significant differences when the genders were combined for analysis (All; P = 0.2 Student's t-test). Two-way analysis indicated that CGRP transcript levels did not differ between genotypes within a gender, or between genders within a genotype (P>0.2 each factor). Figure 6B illustrates levels of RAMP1 mRNA in the spinal cord of *Nf1^+/−^* and WT littermates (Fig. 6B). Levels of RAMP1 transcript did not differ between genotypes when genders were combined for analysis (All; P>0.1; Student's t-test). Two-way analysis indicated that RAMP1 transcript levels did not differ between genotypes within a gender, or between genders within a genotype (P>0.05 each factor). The difference between male *Nf1^+/−^* and WT mice was not statistically significant (P = 0.1; Student's t-test).

## Discussion

This extensive characterization of the nociceptive responses of male and female *Nf1^+/−^* mice was prompted by (1) the enhanced excitability of primary afferent neurons in *Nf1^+/−^* mice [16], (2) the increased release of CGRP from sensory neurons of *Nf1^+/−^* mice [14], and (3) the well-established role of CGRP as a nociceptive neurotransmitter in the periphery and spinal cord [9]–[12]. The results indicate that *Nf1^+/−^* mice did not differ from WT mice in responsiveness to acute heat stimuli delivered at a rate that preferentially activated either Aδ- or C-fibers [27], [28]. They also did not differ from WT mice in responsiveness to innocuous mechanical stimuli with the exception of a subtle enhanced mechanical sensitivity in female *Nf1^+/−^* mice. Given that neither content nor the basal release of CGRP from cultured DRG neurons and spinal cord slices differed between WT and *Nf1^+/−^* mice [14], it is perhaps not unexpected that WT and *Nf1^+/−^* mice did not differ in their responsiveness to brief heat or mechanical stimuli in the absence of inflammation. O'Brien et al. reached a similar conclusion in their recent survey of the responsiveness of *Nf1^+/−^* mice to noxious heat or itch-producing stimuli [21].

### Nociceptive phenotype in inflammatory models

Intraplantar injection of capsaicin releases CGRP from the central and peripheral terminals of primary afferent neurons [14], [30]–[33]. Neither female nor male *Nf1^+/−^* mice differed from their WT littermates with respect to heat hyperalgesia induced by ipl capsaicin. Female *Nf1^+/−^* mice also did not differ from their WT littermates in the magnitude of mechanical hypersensitivity that developed, and male *Nf1^+/−^* mice exhibited only slightly less mechanical hypersensitivity than WT littermates. These findings were unexpected given that capsaicin evokes greater release of CGRP from the terminals of nociceptive afferents in *Nf1^+/−^* mice than WT mice [14].

The formalin test was used to assess nociceptive phenotype in a model of more prolonged inflammation and as a non-reflexive measure of nociceptive behaviors. Formalin-evoked pain behaviors are also dependent on CGRP [34]. Formalin directly activates transient receptor potential (TRP), subfamily A, member 1 channels (TRPA1) [35], [36], and TRP channel, subfamily V, member 1 (TRPV1) channels [37] in DRG neurons. In mice, many TRPV1-immunoreactive primary afferent neurons coexpress TRPA1 [38], [39]. Formalin is therefore likely to cause a central and peripheral release of CGRP similar to that caused by capsaicin. Indeed, male *Nf1^+/−^* mice did not differ from WT littermates in either the duration of licking or other nociceptive behaviors in agreement with the report by O'Brien et al. [21]. Female *Nf1^+/−^* mice showed less licking than WT mice, but this observation is not necessarily indicative of a reduced nociception because these mice also exhibited additional competing behaviors that suggested that formalin was more noxious.

Subsequent experiments injected CGRP into the hindpaw to limit the site of action to the periphery. A more consistent phenotype of exacerbated nociception emerged in this model. Both male and female *Nf1^+/−^* mice exhibited greater heat hyperalgesia than their respective WT littermates after ipl injection of CGRP. Male *Nf1^+/−^* mice also exhibited greater mechanical hypersensitivity their WT littermates, although female *Nf1^+/−^* mice exhibited less mechanical hypersensitivity than their WT littermates. It is well established that CGRP increases its own synthesis, and most likely its own release from sensory neurons [20], [40]. Although levels of transcript (this study) and protein [14] for CGRP were equivalent in the DRG of *Nf1^+/−^* and WT mice, an enhanced release of endogenous CGRP from the peripheral terminals of primary afferents in *Nf1^+/−^* mice cannot be excluded. Another mechanism that may be responsible for the enhanced nociceptive effects of peripherally administered CGRP in *Nf1^+/−^* mice involves invading macrophages and the subsequent release of inflammatory cytokines [41]. Macrophages in *Nf1^+/−^* mice may express increased numbers of the CGRP receptor or RAMP1, or receptors of higher affinity or efficacy upon activation leading to increased release of cytokines. Additional studies will be required to test this hypothesis.

### Nociceptive Phenotypes of Other Ras-GAP Deficient Mice

Recently, the nociceptive phenotype of another Ras-GAP deficient mouse was investigated. Mice with a heterozygous mutation for Synaptic GAP (SynGAP), a neuronal Ras-GAP, also did not differ from WT mice in their responsiveness to heat or mechanical stimuli in the absence of inflammation [42]. As observed with *Nf1^+/−^* mice, ipl injection of capsaicin induced equivalent mechanical hypersensitivity in *SynGAP* deficient and WT mice [42]. Although capsaicin induced greater heat hyperalgesia in *SynGAP* deficient mice than WT mice, this result can be attributed to the finding that *SynGAP* mice have three-fold higher levels of TRPV1 in the DRG [42]. It is not known whether the DRG of *Nf1^+/−^* mice have higher levels of TRPV1 than WT mice. However, given that the magnitude of capsaicin-induced heat hyperalgesia was similar in both genotypes, this is considered unlikely.

## Conclusions

The results of this study do not support the hypothesis that a reduction in neurofibromin is associated with enhanced acute or inflammatory nociception, and confirm the conclusions of another recent comprehensive analysis of male *Nf1^+/−^* mice. [21]. The present study extends this conclusion to additional models of inflammatory injury and also includes female *Nf1^+/−^* mice. Gender is an important consideration given that many chronic pain conditions such as migraine and fibromyalgia are more prevalent in women than men [43], [44].

This study also provides new information relevant to the ‘CGRP hypothesis’ posited by Hingtgen and colleagues. It determined that levels of transcript for CGRP were unchanged in the DRG of *Nf1^+/−^* mice, as were levels of transcript for RAMP1 in the spinal cord. The finding of enhanced heat hyperalgesia in both genders and of mechanical hypersensitivity in male *Nf1^+/−^* mice after ipl injection of CGRP suggests that the peripheral actions of CGRP may be enhanced as a result of neurofibromin deficit. This exacerbation may be the result of increased peripheral release of neurotransmitter from primary afferent neurons or the expression of CGRP receptors by invading macrophages that release proinflammatory cytokines in response to CGRP. As noted earlier, high densities of CGRP-immunoreactive fibers are present in neurofibromas in patients [13]. Thus, consideration should be given to testing the efficacy of CGRP receptor antagonists developed for relief of migraine [45] in diminishing bodily pain and pain associated with nerve sheath tumors in NF patients.
